# Sugarcane Straw as a Source of Arabinoxylans: Optimization and Economic Viability of a Two-Step Alkaline Extraction

**DOI:** 10.3390/foods12122280

**Published:** 2023-06-06

**Authors:** Joana R. Costa, Maria J. Pereira, Sílvia S. Pedrosa, Beatriz Gullón, Nelson M. de Carvalho, Manuela E. Pintado, Ana Raquel Madureira

**Affiliations:** 1 CBQF–Centro de Biotecnologia e Química Fina–Laboratório Associado, Escola Superior de Biotecnologia, Universidade Católica Portuguesa, Rua de Diogo Botelho 1327, 4169–005 Porto, Portugal; jrcosta@ucp.pt (J.R.C.); mjvpereira@ucp.pt (M.J.P.); ncarvalho@ucp.pt (N.M.d.C.); mpintado@ucp.pt (M.E.P.); rmadureira@ucp.pt (A.R.M.); 2 Departamento de Enxeñaría Química, Facultade de Ciencias, Universidade de Vigo, 32004 Ourense, Spain; bgullon@uvigo.es

**Keywords:** sugarcane straws, arabinoxylan, alkaline extraction, response surface methodology, economic viability

## Abstract

Sugarcane processing produces a significant amount of byproducts in the form of straw and bagasse, which are rich in cellulose, hemicellulose, and lignin. This work aims to provide a valorization approach to sugarcane straw by optimizing a two-step alkaline extraction of arabinoxylans by a response surface methodology to evaluate a potential industrial-scale production. Sugarcane straws were delignified using an alkaline–sulfite pretreatment, followed by alkaline extraction and precipitation of arabinoxylan, a two-step process optimized using a response surface methodology. A KOH concentration of (2.93–17.1%) and temperature (18.8–61.2 °C) were chosen as independent variables, and the arabinoxylan yield (%) as a response variable. The model application shows that KOH concentration, temperature, and the interaction between both independent variables are significant in extracting arabinoxylans from straw. The best-performing condition was further characterized by FTIR, DSC, and chemical and molecular weight evaluation. The straws arabinoxylans presented high purities levels, ca. 69.93%, and an average molecular weight of 231 kDa. The overall estimated production cost of arabinoxylan from straw was 0.239 €/g arabinoxylan. This work demonstrates a two-step alkaline extraction of the arabinoxylans method, as well as their chemical characterization and economic viability analysis, that can be used as a model for industrial scale-up production.

## 1. Introduction

Arabinoxylans (AX) present different functional and biological properties, such as water absorption, viscosity-enhancing, emulsifying, gel/film forming abilities, antioxidant capacity, a modulator of the immune response, and regulator of the glycemic index. All these properties show the important value of these compounds and their wide application in different industries, such as food, pharmaceutical or cosmetic applications [1,2]. These compounds can be produced from different sources, including wheat, barley, corn, and other grains such as rice, sorghum, rye, and oat [3].

Sugarcane byproducts such as straw and bagasse represent a promising sources of AXs. Global sugarcane production is expected to rise to 1924 Mton/year of sugarcane by 2031, mainly for sugar production [4]. Sugarcane straw (SCS) is a common agro-industrial composed of dry leaves and tops commonly, that is often burnt as a source of energy for sugar mills [5,6,7], but a major part is thrown in fields following harvesting, emphasizing the need to develop added-value uses for this residue [6,8,9].

Most of the available described extraction processes of AXs are related to the sugarcane bagasse [10,11,12] since this byproduct is brought to the factory and identified as a byproduct of the sugar processing. Nevertheless, SCS is rich in cellulose, hemicellulose, and lignin, presenting low concentrations of extractives and mineral salts [7,13]. Hemicellulose represents 16.8–32.2% of the SCS [14] and is usually composed of β-(1-4)-linked monomeric units of D-xylose, L-arabinose, D-glucose, D-galactose, 4-O-methyl glucuronic acid, D-galacturonic acid, and D-glucuronic acid, and the most common polysaccharides include xylan, arabinoxylan, and xyloglucan [15].

A range of extraction methods has been used to produce AXs, including physical treatments, alkaline, enzyme, and hydrothermal extractions, as well as possible combinations of these approaches [16]. Arabinoxylans contain a chemical backbone comprising xylose residues linked by -(1,4) bonds with arabinose residues substituted at the C(O)-2 and/or C(O)-3 locations. Arabinose’s C(O)-5 position can be used to ester bond phenolic acids such as ferulic acid. In oxidizing conditions, these ferulic acid residues undergo oxidative cross-linking, forming bridges between and within the ferulic acid chains [15,17]. The molecular structure of AXs is influenced by the extraction process; in particular, the alkaline treatment affects the functional groups of AXs, lowers the concentration of ferulic acid, and maintains their original molecular weight [18].

As hemicelluloses are tightly attached in the plant cell to lignin and cellulose, it is difficult to separate them without significantly modifying their structure [19]. Moreover, both hemicelluloses and lignin are soluble in alkaline media, challenging even more the purification of these polysaccharides. There is a lack of information on the major parameters, such as extraction temperature and the solvent concentration in the extraction of AXs from SCS, and an estimation of the costs associated with these processes to envisage their pilot and industrial scale-up. Thus, this work aims to describe a valorization approach of sugarcane straw through the optimization of a two-step alkaline extraction of AXs, by a response surface methodology and their chemical characterization to identify the major differences with other AXs from other extraction methods and an assessment of the economic viability.

## 2. Materials and Methods

### 2.1. Raw Materials

The straws from sugarcane, harvested in October 2018, were provided by Raízen (São Paulo, Brazil). The byproducts were oven-dried at 40 °C for 24 h and milled in a cutting mill (Retsch, Haan, Germany). The extractives were removed with ethanol 50% (*v*/*v*) for 24 h in an orbital incubator at room temperature. The solid fractions were re-dried at 40 °C for 24 h and sieved to achieve particle sizes between 150 and 900 μm. The resulting extractive-free biomasses were characterized for moisture and mineral contents. The lignin, cellulose, and hemicellulose content of extractive-free SCS were determined according to the NREL/TP-510-42618 procedure [20]. The commercial benchmark used to compare with AXs straw was xylan from sugarcane bagasse (Meryer Chemical Technology, Shangai, China).

### 2.2. Fractionation Process of Sugarcane Straw

Lignin was removed from sugarcane straws through alkaline–sulfite extraction. In brief, SCS was mixed with Na_2_SO_3_ 10% (*w*/*w*) solution containing 5% (*w*/*w*) NaOH (LabChem Inc., Zelienople, PA, USA), using a solid-to-liquid ratio (SLR) of 1:15, at 90 °C for 4 h. The insoluble residues of SCS were collected by filtration, thoroughly washed with dH_2_O, and dried at 55 °C.

Arabinoxylans were extracted from delignified SCS biomass through incubation for 16 h at 25 °C with KOH (LabChem Inc., Zelienople, PA, USA) with a liquid/solid ratio of 20:1, followed by filtration under vacuum. For AXs precipitation, 2 V of ethanol (Honeywell, Charlotte, North Carolina, USA) and 0.2 V of glacial acetic acid (Sigma-Aldrich, St. Louis, USA) were added to the solution, incubated at −20 °C for 24 h, and further centrifuged at 4696× *g* for 10 min. The recovered polysaccharide was then freeze-dried and characterized for lignin, hemicellulose, and cellulose content.

#### Experimental Design

A response surface methodology based on a 2^2^ central composite design was proposed to discover the optimal xylan extraction conditions, with AXs extraction yield as a response. According to Table 1, two components were examined as independent variables: solvent concentration and extraction temperature, both of which were studied at five levels.

The time of extraction and liquid:solid ratio was studied using fixed values. An S/L ratio of 20:1 was used based on preliminary studies, corresponding to the minimum amount of water to disperse the biomass, and used by other authors for the alkaline extraction of sugarcane bagasse [21]. The extraction time was defined as a fixed variable; since time and temperature are dependent variables, only temperature was chosen as the optimizable parameter. The time of extraction of 16 h was defined based on the study of Höije et al. (2005), that performed an alkaline extraction during 16 h at room temperature to extract arabinoxylans from barley husks [22].

A KOH concentration (2.93–17.1%, *x*_1_) and temperature (18.8–61.2 °C, *x*_2_) were chosen as independent variables in the current research, with AXE yield (%, y1) as the response variable (Table 1). Preliminary experiments (data not shown) were used to determine the independent variables and their variation ranges.

The following Equation (1) was used to fit the correlations between responses and treatment conditions independent variables to a second-order polynomial model:

(1) $$\gamma_{j\;}=b_{0}+b_{1}x_{1\;}+b_{2}x_{2\;}+b_{11}x_{1}^{2}{}_{}+b_{22}x_{2}^{2}{}_{}+b_{12}x_{1\;}x_{2\;}$$

where *b_n_* are constant regression coefficients, *y* is the response (AX yield), and *x*_1_ and *x*_2_ are the coded independent variables (concentration of potassium hydroxide and process temperature, respectively).

### 2.3. Chemical and Structural Characterization of AXs

#### 2.3.1. Chemical Composition

To quantify the oligosaccharide content in the liquid phase, a post-hydrolysis technique employing sulfuric acid (4% *w*/*w*) at 121 °C for 30 min was performed using the modified National Renewable Energy Laboratory (NREL) approach reported in the literature [20]. An Agilent 1260 Infinity II High-Performance Liquid Chromatograph (HPLC) with a Refractive Index (RI) Detector, an ion-exclusion Aminex HPX-87H (Bio-rad, Berkeley, CA, USA) column at 50 °C, and H_2_SO_4_ 5 mM at an isocratic flow rate of 0.6 mL/min as mobile phase was used to analyze monosaccharides. Using external calibration curves in concentrations between 0.2 and 2.0 mg/mL, each component was identified and quantified by comparison to the retention times of pure standards (glucose, xylose, arabinose, mannose, and galactose) (Sigma Aldrich, St. Louis, MO, USA).

#### 2.3.2. Evaluation of Molecular Weight (MW)

Arabinoxylans were solubilized into an aqueous solution containing 8% (*v*/*v*) of ethanol 99% (Honeywell, St. Louis, MO, USA), stirred at 50 °C for 30 min, and syringe-filtered using 0.45 μm polyester syringe filters (Macherey-Nagel, Düren, Germany). Assessment of MW of AX was performed through size exclusion chromatography (SEC). Briefly, MW was determined using an Agilent 1290 Infinity II HPLC, equipped with an evaporative light scattering detector (ELSD) and a PL aqua gel-OH MIXED-M 7.5 × 300 mm, 8 μm column (Agilent Technologies, Santa Clara, CA, USA). The method was run using 10 mM NH₄CH₃CO₂ at an isocratic flow of 0.6 mL/min as eluent, an evaporation temperature of 80 °C, nebulization temperature of 85 °C, and nitrogen flow of 1.24 SLM. Pullulan P-82 standards Shodex (Showa Denko K. K., Tokyo, Japan), with molecular weights ranging from 5.9 (P-5) to 708 kDa (P-800), were used as an external calibration curve.

#### 2.3.3. Differential Scanning Calorimetry

Employing a differential scanning calorimeter (DSC 204 F1 Netzsch), calorimetric experiments were conducted. The measured samples were sealed in pans made of aluminum. A nitrogen flow of 40 mL.min^−1^ and a linear heating rate of 10 °C/min were used for the studies, which were conducted in the temperature range of −80 to 400 °C. An empty crucible served as the reference sample for the indium-based calibrations of heat flow and temperature.

#### 2.3.4. Fourier-Transform Infrared Spectroscopy

A Spectrum 100 FTIR spectrometer (PerkinElmer, Boston, Massachusetts, USA), equipped with an ATR accessory from PIKE Technologies in the United States and a diamond/Se crystal plate, was used to conduct the attenuated total reflection Fourier transform infrared spectroscopy (ATR-FTIR) investigation. A total of 64 scans were performed, and spectra were gathered with a resolution of 1 cm^−1^ in the absorbance range of 4000 to 400 cm^−1^.

### 2.4. Production Cost Estimation

An economic assessment of the mass and energy costs were used to perform this analysis. A traditional cash-flow analysis was performed using Microsoft Excel software. The major price and costs of the raw materials and equipment are presented in Section 3.4. of the results and discussion. The cost of the initial biomass (sugarcane straw) was not considered since it was assumed it was a residue with no cost associated with its production. Estimated xylan production costs include, in addition to raw materials and energy expenses, overheads (80% of which were examined), waste management, equipment depreciation, personnel costs, and miscellaneous expenditures. Section 3.4. of Estimation of Production costs lists important hypotheses that were utilized to calculate the manufacturing process costs, considering all costs in Euros.

The reagent costs were determined based on market reference values and the experimental mass balance (data not shown). The equipment was selected from industrial suppliers based on their capacity and specifications (e.g., type of material, capacity, and power consumption), and information on their power consumption was used for energy cost estimation (kWh). According to Eurostat for non-household consumers, the electricity price considered was €0.1529 kWh-1 in the first half of 2022. The tap water price considered was €1.9 per m^3^, in accordance with Portugal level 3 prices. We did not consider the costs of equipment acquisition. Results are presented as an estimated cost per 1 kg of biomass processes.

### 2.5. Statistical Analysis

The regression analysis feature of the Data Analysis Add-In (Microsoft Excel, USA) was used to fit experimental data. The lack of fit, the coefficient of determination (R^2^), and the F-test value obtained from the analysis of variance were all evaluated to assess the model’s suitability. 

## 3. Results and discussion

### 3.1. Chemical Composition of Sugarcane Straw Biomass

The chemical composition of the sugarcane straw samples used is expressed as % (*w*/*w*) of raw material ± standard deviation (Table 2). Similar compositions were found by Rocha et al. (2015) [7].

Sugarcane is usually rich in different classes of polysaccharides, hemicellulose, that can compose up to 25% of its structure, including arabinogalactans, xylan and heteroxylans, pectins, dextran or galactomannan [23]. Sugarcane Straw presented a hemicellulose composition of 260.1 ± 3.2 g.kg^−1^ SCS, from which 212.9 ± 1.4 g are structurally composed of xylose monomers and 47.2 ± 1.8 g of arabinose monomers. The hemicellulose content determined in SCS is similar to the ones reported in the literature, such as 27–31% [24] and 31% [25].

After removing extractives (see Table 3), SCS was shown to have a similar carbohydrate profile, with cellulose as the most representative one with 362.9 ± 15.0 g cellulose.kg^−1^ SCS. The total lignin contents were 179.1 ± 0.6 g lignin.kg^−1^ SCS. Overall, these profiles are per the results reported by Costa et al. (2013) [26] for raw sugarcane straw (33.5 wt % cellulose and 21 wt % lignin) [6]. Comparing with other sources, rather than sugarcane, the AX’s levels in different types of cereals range from 2 to 8%. Rye has a value of 5.3–8.4%, barley has an amount of 4–8%, wheat has a content of 4–6%, and oats have a content of 2.2–4.1% of AX’s [27].

Considering the composition of sugarcane straw, the recovery AXs from this source could be profitable and a way to reduce the environmental effect of sugarcane processing.

### 3.2. Extraction Optimization Responses

The delignification treatment of SCS was performed using an alkaline–sulfite pretreatment methodology. This method is based on the fact that alkali treatment disrupts the cell wall dissolving hemicelluloses and lignin by hydrolyzing uronic and acetic esters, and also on the ability of sulfite ions to render lignin sulfonated and therefore more hydrophilic [10]. With mild sulfite pretreatment conditions, it is possible to decrease lignocellulose recalcitrance by increasing its swelling and porosity and, therefore, more easily removing it.

#### 3.2.1. Model Adequacy

The combined central composite design and response surface methodology were used to optimize the values for both independent variables presented in Table 1. Table 4 presents the central composite design matrix of 11 experiments, indicating the different combinations for the independent variables along with the experimental values obtained for the dependent variables (extraction yields). The experimental data were analyzed using multiple regression fitting to obtain linear or quadratic polynomials for extraction yields from SCS. The model’s fit was evaluated by variance analysis (ANOVA). 

Regression coefficients, statistical significance (based on a Student’s *t*-test), and determination coefficient (R^2^) are presented in Table 5. The R^2^ value was 0.945, showing a good correlation between the model and the experimental data for both byproducts. Considering the regression coefficients shown in Table 5, the linear effects of KOH concentration and temperature exerted a significant influence on the AX’s recovery. Additionally, the interaction between KOH concentration and temperature has a significant effect on the extraction yield of AXs. ANOVA analysis showed that the significance level of the model was 88.927, confirming that the developed models are suitable for the prediction of AXs extraction yields.

#### 3.2.2. Response Surface Analysis

Extraction yields of AXs for SCB ranged from 2.21 (Run #8) to 15.37 kg AX/100 kg bagasse (Run #6), while extraction yields for SCS ranged from 4.23 (Run #5) to 16.70 kg AX/100 kg straw (Run #3), as shown in Table 4. These results are in accordance with different authors who reported extraction yields of xylan from sugarcane byproducts using potassium hydroxide, ranging from 6 to 64% [28,29,30]. The AX recovery yields obtained show that the combination of higher temperatures and a lower % of KOH results in lower yields. Additionally, the high temperature used in the delignification pretreatment, which results in the efficient removal of lignin, might compromise the hemicellulose structure [28].

**Figure 1:**
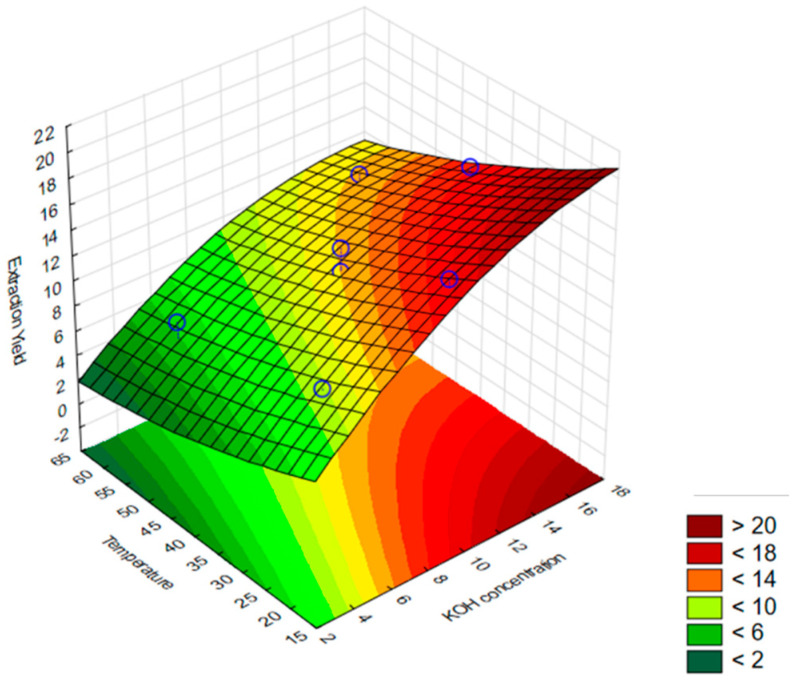
Arabinoxylans extraction yield from SCS, as a function of the KOH concentration and temperature. Blue circles represent the experimental points obtained.

According to Figure 1, the impact of the two variables studied on the extraction yield of AX from SCS is evident. It is clear the positive effect of the KOH concentration on the extraction yields of AX. The increase in temperature shows a negative impact, supported by the negative significant coefficient of temperature (Table 5). Zhao et al. (2021) [30] also found a positive relationship between the potassium concentration and the extraction yield of bagasse but concluded that this parameter was more significant than the incubation temperature. Analyzing the surface response for AX recovery from SCS allowed us to conclude that according to the experimental data obtained in Run #3, which achieves a better extraction yield with a potassium hydroxide concentration of 15% and a process temperature of 25 °C.

### 3.3. Arabinoxylans Properties

#### 3.3.1. Chemical Properties

Although hemicellulose fraction is mainly composed of xylan backbone, several neutral sugar moieties such as arabinose, glucose, or glucuronic acid are also present in different proportions and may vary with the isolation method [28,31]. Table 6 presents the chemical composition of the solids obtained from AXs extraction through alkaline–sulfite treatment. Results show that this method allowed us to obtain an extract rich in AXs, above 60% of purity, with low content in glucose moieties and lignin, like the commercial benchmark (CB). Furthermore, alkaline–sulfite treatment was successful in total lignin contamination from both samples, confirming that this two-step alkaline process is a suitable strategy for obtaining highly purified AXs. The molar ratios Xylose:Arabinose:Mannose:Glucose:Galactose calculated from the compositional data was 10:1.15:0:0.97:0 for sugarcane straw. These results are in accordance with several authors who recovered xylan from sugarcane bagasse with purities of 61.7 ± 0.8 to 64.2 ± 0.2% and from wheat straw with a purity of 66.9% [10,32]. The xylan from sugarcane bagasse used as a benchmark does not have glucan due to a possible purification step which results in a higher purity; however, this would certainly increase the cost of the process.

Overall AXs obtained from straw under optimal extraction conditions have a purity of ca. 60% and are similar to commercial xylan obtained from sugarcane bagasse, except for the presence of glucans.

#### 3.3.2. Structural Properties

Results of commercial AXs and straw AXs molecular weights, evaluated by HPLC-ELDS, are represented in Table 7. As can be observed, Straws AXs has a wide variety of molecular weight (Mw), represented by their high polydispersity, as compared to commercial AXs. The average molecular weight (Mp) of straws AXs ranges from 100 to 200 kDa, 10-fold higher than the molecular weights of glucurono-arabinoxylans recovered from sugarcane bagasse using similar extraction yields but higher alkali concentrations [10]. Results show that although commercial AXs’ Mw is lower, its weighted average (Mn) is higher than the one found in bagasse and straw AXs. Also, commercial AXs’ most representative population (Mp) has a lower MW than the ones of bagasse and straw AXs. It is possible to conclude that although commercial AXs have a less disperse molecular weight distribution, with lower arithmetic molecular weight, higher molecular weight chains are in higher numbers or are larger than the ones in straw AXs (see Mn values). Regarding straws, AXs present higher Mw and weighted average (Mn) molecular weight. 

Differential scanning calorimetry (DSC) analysis presented in Table 8 showed both samples’ endothermic peaks at approximately 90 °C, which may be associated with the onset of hemicellulose degradation indicated by the evaporation of water present in the sample [33]. Both samples suffered total pyrolysis, confirmed by exothermic peaks, with onset temperature at 254.3 °C and placed at 285.3 °C for SCS. Werner et al. (2014) [34] obtained similar thermal behaviour for AXs, with higher degradation temperatures (up to 243 °C). The additional peak was presented in commercial xylan at 310.3 °C, which can correspond to a melting temperature of salt impurities [35].

**Figure 2:**
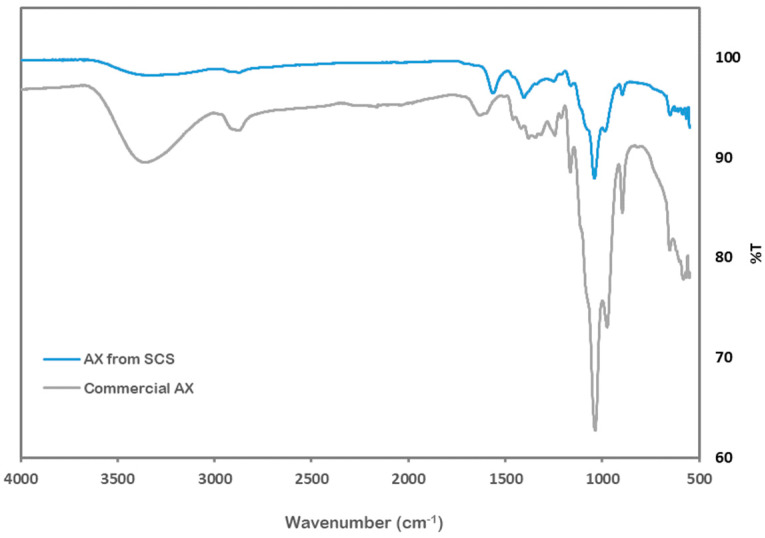
Fourier-transform infrared (FT-IR) spectra of different AXs or commercial benchmarks.

The normalized FT-IR spectra of SCS and commercial AXs (Figure 2) analysis revealed no significant differences between samples. Strong absorption bands were present at 1036 and 983 cm^−1^, attributed to the stretching of CO-O-CO and the C=C bending of the pyranose ring and glycosidic bond, respectively, characteristic of arabinoxylan functional groups [36]. A broad absorption band around 3300 cm^−1^ was also detectable in all spectrums, corresponding to O-H stretching, as well as the intramolecular hydrogen bonds. The bands around 1321 and 1557 cm^−1^ can be assigned to C-H symmetric and asymmetric stretching vibrations within the xylose monomers, respectively.

### 3.4. Estimation of Production Cost

**Figure 3:**
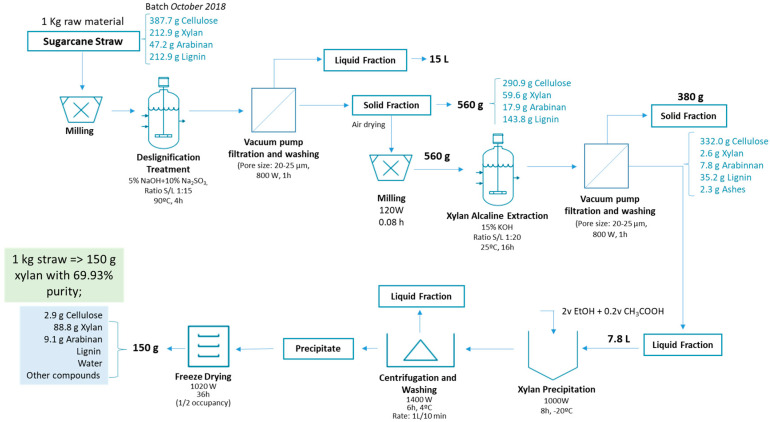
Arabinoxylan extraction from sugarcane straw diagram.

An economic assessment was performed of the integrated process to obtain arabinoxylan from sugarcane straws, comprising the main processing steps: (1) delignification treatment and (2) xylan alkaline extraction. The comprehensive analysis of the economic viability of large-scale xylan production was based on experimental data obtained at a laboratory scale. The optimized values of the operating conditions and the main results obtained in a study performed at a laboratory scale are resumed in lab processes’ mass balances for SCS (in Figure 3).

The overall estimated production cost of arabinoxylan (Table 9) was 0.239 €/g xylan. The largest expense was the absolute ethanol, representing ca. 35% of the total cost. However, at a higher-level production scale, this cost might be mitigated through the recovery and re-utilization of ethanol. The mass balances were calculated at the lab size, but costs for chemicals were based on bulk quantity prices to make this economic simulation as realistic as feasible. Lab-scale procedures are typically more susceptible to lower yields, whether due to mass losses during the process or due to the decreased yield of particular lab equipment.

## 4. Conclusions

Arabinoxylan was successfully extracted from sugarcane straw through a multi-step protocol with high yield and low content in glucan and arabinan. The obtained polysaccharides presented high purities of 69.93% AXs and an average molecular weight of 231 kDa. Moreover, a thermal analysis of both polysaccharides showed a stability of up to 285 °C, confirming the ability of these ingredients to be applied in different industrial processes, including thermal processing. The overall estimated production cost of arabinoxylan from straw was 0.239 €/g xylan. Overall, this work demonstrates that sugarcane byproducts can be a source of sustainable, functional ingredients for a wide spectrum of applications.

## Figures and Tables

**Table 1:** Experimental variables involved in the design.

Variable	Definition and Units	Nomenclature	Value or Range
Fixed	Liquid: solid ratio of extraction (*w*/*w*) Time (h)	LSR	20:1 16
Independent	KOH (%) Temperature (°C)	%KOH T_E_	2.93–17.1% 18.8–61.2 °C
Dependent	Xylan Yield	XY	Kg xylan/100 kg RM

**Table 2:** Composition of sugarcane straws on structural carbohydrates.

Biomass Composition					
Cellulose (g/100 g)	Xylan (g/100 g)	Arabinan (g/100 g)	Lignin (g/100 g)	Arabinan (g/100 g)	Humidity (g/100 g)
38.77 ± 0.43	21.29 ± 0.14	4.72 ± 0.18	20.22 ± 0.78	2.67 ± 0.07	6.15 ± 0.28

**Table 3:** Structural carbohydrates (mean ± S.D.) of SCS biomass after extractives removal.

Biomass Composition					
Cellulose (g/100 g)	Xylan (g/100 g)	Arabinan (g/100 g)	Lignin (g/100 g)	Arabinan (g/100 g)	Humidity (g/100 g)
36.29 ± 1.50	21.64 ± 0.99	3.19 ± 0.26	17.91 ± 0.06	1.41 ± 0.06	7.14 ± 0.50

**Table 4:** Operational conditions are expressed as dimensional and dimensionless independent variables and the response of dependent variable *y_1_*.

Run	KOH (%, *w*/*w*) *x_1_*	Temperature (°C) *x_2_*	Ext. Yield (kg_AX_/100 kg _SCS_)
1	5 (−1)	25 (−1)	9.88
2	5 (−1)	55 (+1)	7.20
3	15 (+1)	25 (−1)	16.70
4	15 (+1)	55 (+1)	12.65
5	2.93 (−1.41)	40 (0)	4.23
6	17.1 (+1.41)	40 (0)	15.51
7	10 (0)	18.79 (−1.41)	16.51
8	10 (0)	61.21 (+1.41)	8.37
9	10 (0)	40 (0)	11.71
10	10 (0)	40 (0)	13.59
11	10 (0)	40 (0)	10.91

**Table 5:** Regression coefficients and statistical parameters used to evaluate model correlation and significance.

Coefficient	SCS
b_0_	12.0687 ^a^
b_1_	7.0551 ^a^
b_2_	−4.5607 ^a^
b_11_	−1.9732
b_22_	0.5980
b_12_	−0.6850
R^2^	0.945
Significance level (%)	88.927

^a^ Significant coefficient at the 95% confidence level

**Table 6:** Structural carbohydrates (mean ± SD) of AXs and commercial benchmark.

Sample	Glucan % (*w*/*w*)	Xylan % (*w*/*w*)	Arabinan % (*w*/*w*)	Lignin % (*w*/*w*)	Purity AX %
AXs	4.50 ± 0.38	59.2 ± 5.60	6.07 ± 1.12	5.43 ± 0.43	65.27
Benchmark	-	73.05 ± 2.34	6.04 ± 0.96	5.00 ± 0.50	79.06

**Table 7:** Molecular weight (mean ± S.D.) of straw AXs compared with commercial xylan.

Xylan	Mp (kDa)	Mw (kDa)	Mn (kDa)	DP
Straw	55.4	715	231	3.09
Commercial	47.7	515	295	1.75

Mp—peak molecular weight; Mw—arithmetic average molecular weight; Mn—number average molecular weight; DP—polydispersity index.

**Table 8:** Summary of DSC peak temperatures of straw and commercial xylan.

Xylan	Peak	Onset (°C)	Temperature (°C)	Energy (J/g)
Straw	1	52.5	88.4	−187.3
	2	254.3	285.3	353.7
Commercial	1	47.9	95.2	−625.7
	2	261.5	297.0	38.17
	3	306.4	310.3	7.89

**Table 9:** Cost balances with a calculation based on 1 kg of raw biomass. General costs are for electricity costs (€/kW.h) of 0.1529 and tap water (€/L) of 0.0019.

**Step 1:** Delignification 5% NaOH and 10% Na_2_SO3				
Equipment	Power (kW)	Time (h)	Electricity Consumption (kW.h)	Costs (€)
Coffee grinder	0.12	0.16	0.0192	0.00
Water bath	2	4	8	1.22
Vacuum pump	0.08	1	0.08	0.01
Reagents	Quantity (kg)	Price (€/kg)		Cost (€)
Sodium Sulphite	0.1	0.33		0.03
Sodium Hydroxide	0.05	0.32		0.02
dH_2_O (solvent)	20	0.18		3.60
Tap Water (washing)	71	0.0019		0.13
**Step 2:** Xylan Extraction with KOH 15% and ethanolic precipitation.				
Equipment	Power (kW)	Time (h)	Electricity Consumption (kW.h)	Costs (€)
Coffee grinder	0.12	0.08	0.0096	0.00
Water bath	2	16	32	4.89
Vacuum pump	0.08	0.5	0.04	0.01
Centrifuge	1.4	6	8.4	1.28
Freezer	1.01	8	8.08	1.24
Freeze-dryer (1/2 occupancy)	0.5	36	18	2.75
Reagents	Quantity (kg)	Price (€/kg)		Cost (€)
Potassium Hydroxide	2.85	0.54		1.54
Absolute Ethanol	21.5	0.6		12.90
Glacial Acetic acid	1.4	0.54		0.76
dH_2_O (solvent)	30	0.18		5.40
Tap Water (washing)	28	0.0019		0.05
Total Costs (€/Kg Biomass)				35.84

## Data Availability

The data presented in this study are available on request from the corresponding author.
